# Cell-Free DNA as a Diagnostic and Prognostic Biomarker in Dogs With Tumors

**DOI:** 10.3389/fvets.2021.735682

**Published:** 2021-09-16

**Authors:** Jihu Kim, Hyeona Bae, Soomin Ahn, Sunwoo Shin, ARom Cho, Kyu-Woan Cho, Dong-In Jung, DoHyeon Yu

**Affiliations:** College of Veterinary Medicine, Gyeongsang National University, Jinju, South Korea

**Keywords:** dog, cell-free DNA, tumor, lymphoid neoplasia, biomarker, prognosis

## Abstract

Cell-free DNA (cfDNA) is derived from apoptosis/necrosis, active cellular secretion, and lysis of circulating cancer cells or micrometastases. In humans, cfDNA is widely used in cancer diagnosis, but veterinary research has yet to be actively conducted to establish it as a cancer biomarker. This retrospective study analyzed cfDNA levels in samples collected from dogs with neoplastic disease (*n* = 38), clinically ill dogs without neoplasia (*n* = 47), and healthy dogs (*n* = 35). cfDNA levels and clinical data were compared among groups, and prognostic analyses were performed within the neoplastic group. Furthermore, continual cfDNA measurements were performed during the chemotherapy of six dogs with lymphoma. Dogs with neoplasia showed significantly higher cfDNA concentrations than dogs without neoplasm, and the cfDNA oncentration in the lymphoid neoplasia group was significantly elevated among all neoplastic groups. Dogs with neoplasia and a plasma cfDNA concentration above 1,247.5 μg/L had shorter survival rates than those with levels below this threshold (26.5 vs. 86.1%, respectively, *P* < 0.05). In cases with complete remission in response to chemotherapy, the cfDNA concentration was significantly decreased compared with the first visit, whereas the cfDNA concentration was increased in cases with disease progression or death. Interestingly, a significant correlation was found between lymph node diameter and cfDNA concentration in dogs with multicentric lymphoma (*R*^2^ = 0.26, *P* < 0.01). These data suggest that changes in cfDNA concentration could be used as a diagnostic biomarker for canine neoplasia. Furthermore, increased plasma DNA levels might be associated with shorter survival time, and cfDNA concentrations may reflect the response to chemotherapy.

## Introduction

Cell-free DNA (cfDNA) is a short double-stranded DNA fragment. A fraction of the cfDNA present in the plasma of cancer patients is derived from cancer cells have been demonstrated (1). Follow-up studies confirmed that cancer cells release detectable amounts of cfDNA fragments into circulation and other body fluids, such as urine, cerebrospinal fluid, pleural fluid, and saliva and revealed that these fragments bear unique genetic and epigenetic alterations characteristic of the tumor of origin (2). Low levels of cfDNA can be detected in healthy individuals. However, higher levels have been detected in human patients diagnosed with some diseases, including cancer. Nearly 50% of all cancer patients have increased levels of circulating cfDNA, and human medicine actively uses cfDNA as a biomarker for early cancer diagnosis, disease staging, and patient prognosis (3), as well as for the detection of minimal residual disease and the prediction of recurrence (4). This increase is seen in multiple tumor types, including hematopoietic tumors (e.g., lymphoma and leukemia), carcinomas (e.g., lung, breast, cervical, pancreatic, and gastrointestinal tumors), and other tumor types, such as sarcoma, melanoma, and glioma (3). Furthermore, many of the same genetic and epigenetic changes occur in circulating DNA and in DNA derived from primary tumors, including mutations and/or aberrant promoter hypermethylation involving *p*53, *p*16, and APC genes. These findings indicate that at least a portion of the circulating DNA is derived from tumor cells (3). In veterinary medicine, increased blood cfDNA concentrations have also been documented in immune-mediated hemolytic anemia (5), sepsis, gastric dilation-volvulus syndrome, and trauma in dogs (6–8). However, the number of studies comparing plasma cfDNA concentrations in dogs with tumors is limited (3, 6, 9). This study aimed to determine whether cfDNA can be used to distinguish dogs with tumors from healthy dogs or dogs with non-malignant diseases. Furthermore, the correlation between cfDNA concentration and survival rate was analyzed. Finally, the role of cfDNA as a therapeutic indicator of the clinical outcome and treatment response in dogs was evaluated.

## Materials and Methods

In this retrospective study, 120 dogs attending the Veterinary Medical Teaching Hospital from 2019 to 2020 were randomly selected and classified into neoplastic (*n* = 38), non-neoplastic (*n* = 47), and healthy (*n* = 35) groups according to their medical records. The study was approved by the Institutional Animal Care and Committee (GNU-210107-T0001).

Control samples were collected from 35 clinically healthy client-owned dogs visiting the hospital for wellness checks, vaccinations, or spays/neuters. Exclusion criteria were a current disease, abnormalities detected on routine clinical examination, and significant abnormalities in laboratory analyses or diagnostic imaging.

The non-neoplastic group included dogs exhibiting clinical symptoms and with a diagnosis other than tumors. Diagnostic confirmation of the non-neoplastic group was made as follows: endocarditis was diagnosed based on echocardiography and blood cultures; hemoparasites such as *Mycoplasma haemocanis* and *Babesia canis* were diagnosed according to polymerase chain reaction (PCR) results; Immune-mediated hemolytic anemia was confirmed based on auto-agglutination tests and spherocytosis in blood film examination; hyperadrenocorticism in dogs was diagnosed based on low-dose dexamethasone suppression tests or adrenocorticotropic hormone stimulation test; pancreatitis was diagnosed based on clinical, SNAP canine pancreatic lipase test (Canine SNAP® cPL^TM^; IDEXX Laboratories) and abdominal ultrasound; intestinal lymphangiectasia and vascular ectasia were confirmed based on gross lesions on gastrointestinal endoscopy and histopathology of biopsy samples; finally, bronchomalacia and atherosclerosis were diagnosed based on computed tomography scans (Canon Aquilion Lightning 160; Canon Medical Systems Corporation, Otawara, Japan). Dogs with a history of exogenous steroid treatment were excluded from this study.

The tumor types in the neoplastic group varied but were classified into one neoplastic group comprising 38 dogs with malignancies. The definitive diagnosis was established by histopathology, immunophenotyping by flow cytometry, or PCR for antigen receptor rearrangement in cases of lymphoma (by Colorado State University via IDEXX Laboratories). The sum of superficial lymph node diameter (LD) with multicentric lymphoma was measured by two veterinarians based on existing consensus (10). The malignancies were staged depending on the type of tumor, according to the World Health Organization staging system (11) based on computed tomography, abdominal ultrasound, and hematologic examinations. In terms of clinical outcomes, the observation times were calculated immediately following the diagnosis. Serial blood collections were performed on six dogs with lymphoma to determine whether cfDNA levels were correlated with disease progression. The criteria for response to treatment and relapse were defined as described for lymphomas in (10) and evaluated at every visit.

Blood samples were collected via jugular venipuncture and stored in EDTA tubes for cfDNA analysis. Supernatants following centrifugation of plasma samples for 5 min at 2,000 × *g* were stored at −80°C until further use. To obtain optimal cell-free plasma, samples were thawed at room temperature for 10 min, followed by centrifugation for 10 min at 16,000 × *g* and 4°C (12). Only purified supernatants after centrifugation were used for cfDNA analyses. The cfDNA was quantified using the Qubit dsDNA HS Assay Kit and a Qubit 1.0 fluorometer (Life Sciences, Carlsbad, CA, USA) according to the manufacturer's specifications. The Qubit assay utilizes a dye that fluoresces with a higher intensity when bound to dsDNA, and the recorded fluorescence intensity is proportional to the amount of dsDNA within the sample (13). The concentration of cfDNA was calculated using the dilution algorithm provided by the manufacturer within the Qubit 1.0 fluorometer (Life Sciences). The Qubit 1.0 fluorometer was calibrated with the specified standards before each measurement. All samples were assayed in duplicate.

For statistical analyses, normality was assessed using the Shapiro-Wilk test. Mann-Whitney U test was used to compare cfDNA mean concentrations by dividing the groups into two: healthy and clinically ill dogs. After dividing the groups into healthy, non-neoplastic illness, and neoplastic, the Kruskal-Wallis test was used to compare the mean concentration of cfDNA and age of the three groups. The mean concentration of cfDNA after dividing it into six groups according to the origin of tumor was compared using ANOVA. The post-analysis method was used for the Dunnett *T3* test. All statistical models were corrected for fixed effects of age and sex by multiple regression analysis. The diagnostic value of cfDNA for predicting survival time was assessed using a receiver operating characteristic (ROC) curve (14). All blood samples used to assess the association between survival rate and cfDNA were collected at the time of diagnosis. During the 60 weeks, dogs that died from tumor progression were classified as death (uncensored) data. In addition, follow-up loss, death from other causes aside tumors, and data from survivor dogs without events during the observation period were classified as censored data. When the cut-off value was obtained, the ROC curve was drawn by setting the test variable to cfDNA and death by the tumor progression variable during the observation period. Hence, the cut-off value with the highest sensitivity and specificity was obtained. The Kaplan-Meier curve analysis was used to determine the survival time of the tumor group with the cutoff value obtained from the ROC curve analysis. During the survival analysis, dogs euthanized due to disease progression were included in the non-survival group. In cases with multicentric lymphoma, Spearman's correlation test was used to correlate the sum of LD with DNA concentrations. Statistical significance levels were set at *P* < 0.05 and *P* < 0.01. The statistical software package SPSS (SPSS 25.0; SPSS Inc., Chicago, IL, USA) was used to perform all statistical evaluations. All graphs were plotted using GraphPad Prism 7.0 (GraphPad Software, La Jolla, CA, USA).

## Results

### Classification and Characterization of the Study Population

The enrolled 35 healthy dogs included 21 males (4 intact) and 14 females (4 intact), with a median age of 5.4 years (range: 1–19 years). The group of 47 dogs with non-neoplastic diseases comprised 26 males (4 intact) and 21 females (9 intact), with a median age of 9.5 years (range: 1–17 years), whereas the 38 dogs with neoplasms included 11 neutered males and 27 females (4 intact), with a median age of 11.0 years (range: 3–20 years). The age values of symptomatic dogs with and without neoplasms were significantly higher than that of healthy control dogs (*P* < 0.01 and *P* < 0.05, respectively). The most common malignant tumor types according to the origin of the tumors were carcinoma (*n* = 15; three dogs with hepatocellular carcinoma, three dogs with ectopic thyroid carcinoma, two dogs with pulmonary adenocarcinoma, two dogs with mammary carcinoma, 1 dog with thyroid C-cell carcinoma, one dog with squamous cell carcinoma, one dog with renal cell carcinoma, one dog with transitional cell carcinoma, and one dog with intestinal adenocarcinoma), lymphoid neoplasia (*n* = 13), sarcoma (*n* = 5; 1 dog with hemangiosarcoma, one dog with stromal sarcoma, one dog with oral leiomyosarcoma, one dog with gastrointestinal stromal tumor, and one dog with multilobular osteochondrosarcoma), mast cell tumor (*n* = 2), and 1 each of pheochromocytoma and peripheral nerve sheath tumor. Signalment characteristics of each group are listed in Supplementary Table 1.

### Comparison of Cell-Free DNA Concentrations

**Figure 1:**
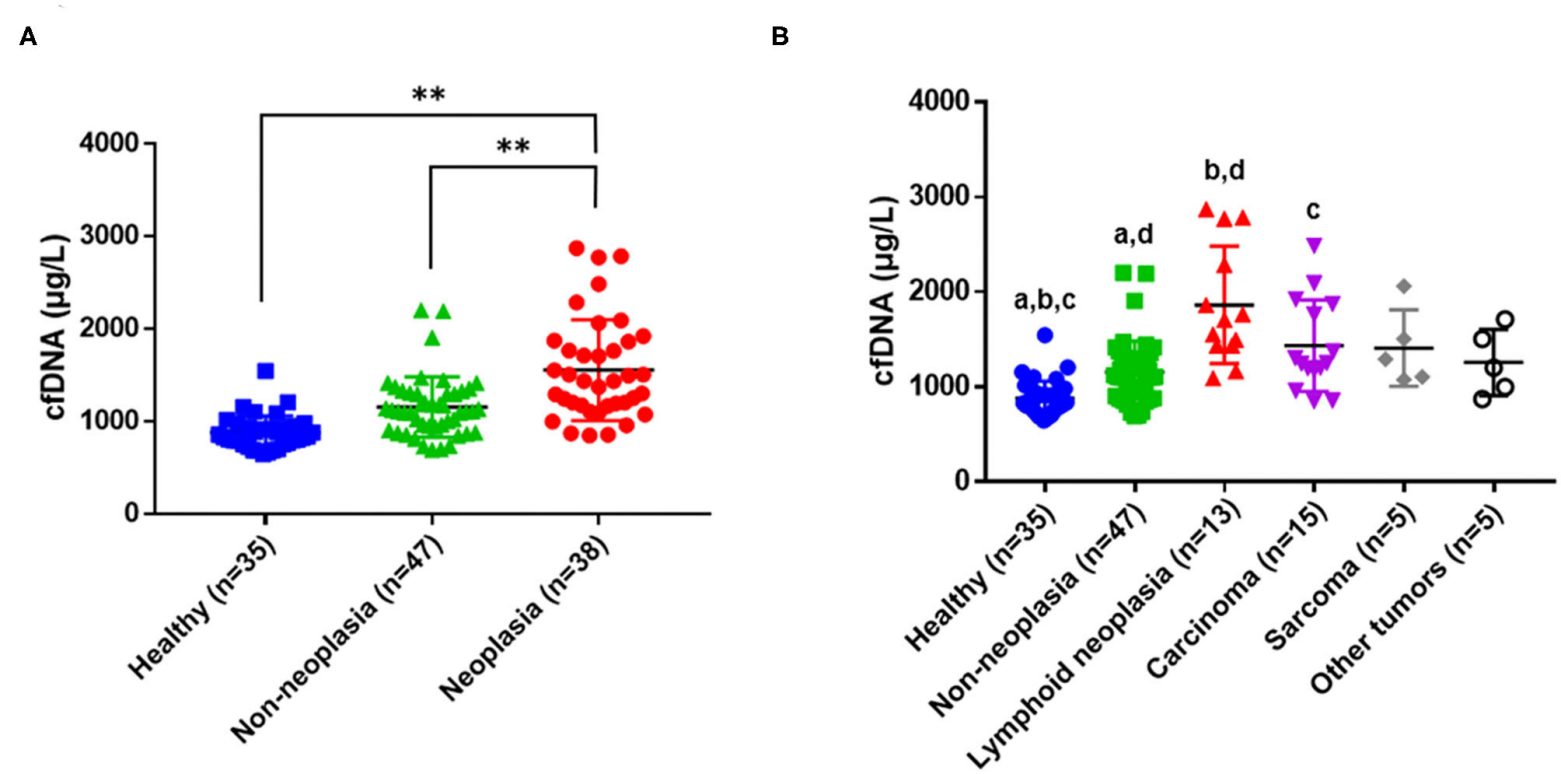
Concentrations of cell-free DNA (cfDNA) in healthy dogs, dogs with underlying non-neoplastic diseases, and dogs with tumors. **(A)** Comparison of mean cfDNA concentrations among the healthy, non-neoplastic, and neoplastic groups (^**^P < 0.001 between groups). **(B)** Horizontal lines inside a plot indicate median values, and whiskers indicate interquartile ranges. The same letters (a, b, c, *P* < 0.01; d, *P* < 0.05) indicate a significant difference between a pair of data.

The mean cfDNA concentration in clinically ill dogs (from both neoplastic and non-neoplastic groups) was 1,333 ± 478.3 μg/L, which was significantly higher (*P* < 0.001) than that in healthy dogs (877.8 ± 181.7 μg/L). When clinically ill dogs were subdivided into neoplastic and non-neoplastic groups, the mean cfDNA concentration in the neoplastic group was significantly different from those in the non-neoplastic and healthy groups (both *P* < 0.001; Figure 1A). The mean cfDNA concentrations in dogs with and without neoplasms were 1,553.9 ± 544.3 μg/L and 1,154.4 ± 326.5 μg/L, respectively. When the neoplastic group was subclassified according to tumor origin [lymphoid neoplasia (*n* = 13), carcinoma (*n* = 15), sarcoma (*n* = 5), and other tumors (*n* = 5)], dogs with lymphoid neoplasia had significantly higher plasma cfDNA levels (Figure 1B). Within the lymphoid neoplasia group, the mean cfDNA concentrations in tumors of *B*-cell (*n* = 5) and *T*-cell (*n* = 5) origin were 1,594 and 2,097 μg/L, respectively. The remaining three dogs in this group are non-*B* non-*T* (*n* = 2) or concurrent *B* and *T*-cell (*n* = 1) origins, and their mean cfDNA concentrations were at 2,025 and 1,705 μg/L, respectively.

### Diagnostic and Prognostic Evaluation of Dogs With Tumors

**Figure 2:**
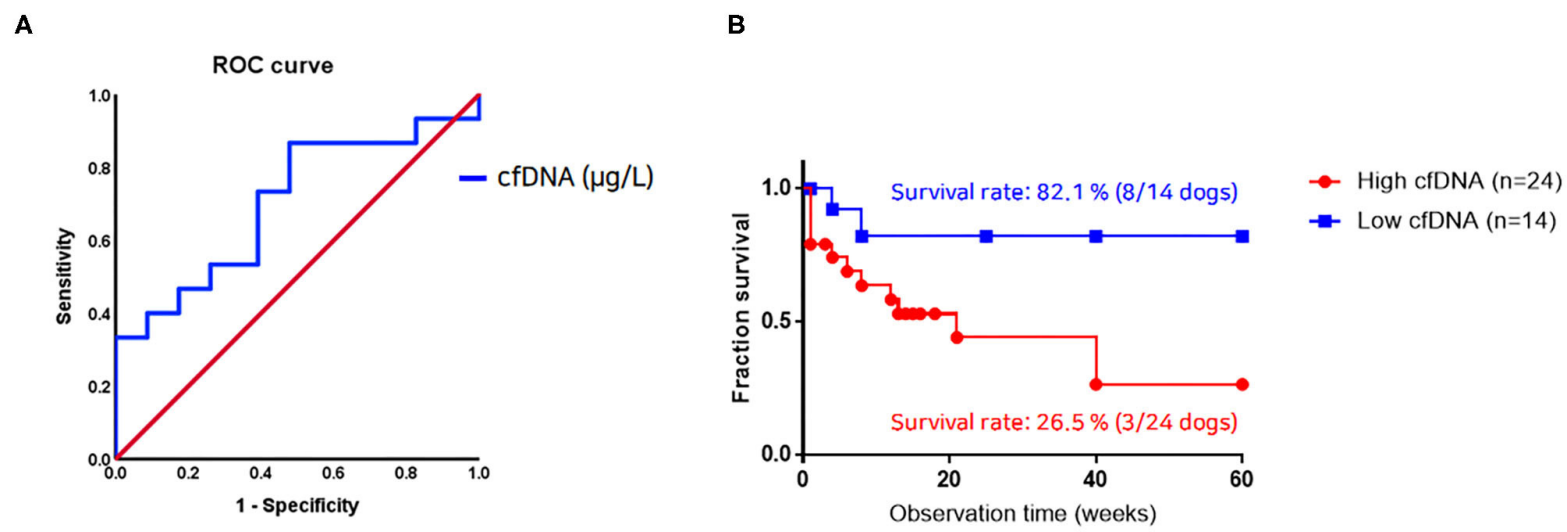
Relationship between cell-free DNA (cfDNA) level and survival rate in dogs with neoplastic diseases. **(A)** Receiver operating characteristic curve analysis identifying diagnostic sensitivity and 1-specificity of cfDNA levels. The area under the curve (95% confidence intervals) is 0.701 (0.523–0.880; *P* < 0.05). **(B)** The Kaplan-Meier plot using a cfDNA concentration of 1,247.5 μg/L as the cutoff shows a statistically significant difference (*n* = 38, *P* < 0.05). A dog from the neoplastic group with a cfDNA concentration above this cutoff value has a survival rate of 26.5% at the time of tumor diagnosis. By contrast, dogs from this group with a cfDNA value below this cutoff value have a survival rate of 82.1%.

The plasma cfDNA of the 38 dogs with neoplasms and the disease progression in the follow-up period were compared to assess the prognostic significance of increased cfDNA levels (Figure 2A). Based on a cutoff value of 1,247.5 μg/L, dogs with tumors were divided into those with a cfDNA concentration of ≤ 1,247.5 μg/L (14 dogs) and those with a cfDNA concentration of >1,247.5 μg/L (24 dogs). In the ROC curve analysis, the area under the curve was 0.701 (95% confidence interval, 0.523–0.880; *P* < 0.05), and sensitivity and specificity were 86.7 and 52.2%, respectively. When dogs in the neoplastic group were monitored for 60 weeks, the probability of survival varied depending on the DNA concentration. Treatment and survival were not significantly correlated (*P* = 0.120) during the time of observation. However, the cfDNA concentration on admission significantly affected the survival rate. The survival rate was 82.1% for dogs with low cfDNA concentrations but only 26.5% for those with high cfDNA concentrations (Figure 2B). Although the positive predictive value of this test as a diagnostic or prognostic indicator was only 54.2%, its negative predictive value was 85.7%.

### Monitoring of Therapeutic Responses Using cfDNA Levels

**Figure 3:**
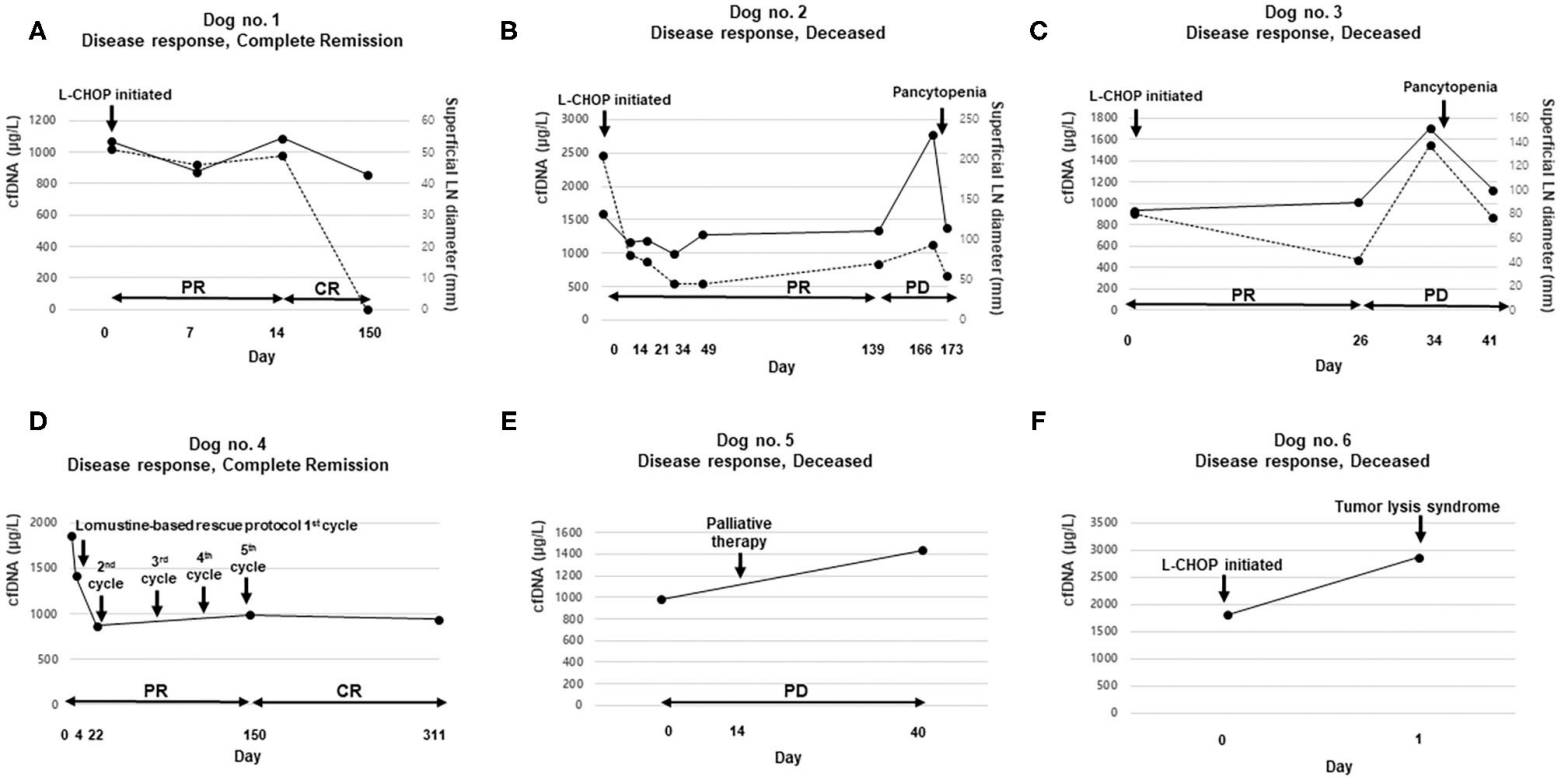
Comparison of cell-free DNA (cfDNA) concentration according to treatment response: cfDNA level (solid line) and the sum of lymph node diameter (LD) values (dotted line) at various time points in six dogs. The left and right vertical axes represent cfDNA concentrations and the sum of LD, respectively **(A–C)**. The horizontal axes represent the days of plasma sampling and the tumor response status **(A–F)**. The detailed signalments of the dogs are provided in Supplementary Table 2 **(A,B,C,F)**. Dogs with multicentric *B*-cell lymphoma treated with the UW-Madison-19 (L-CHOP) protocol; **(D)** a dog with multicentric *T*-cell lymphoma treated according to a lomustine-based rescue protocol; and **(E)** a dog with non-epitheliotropic cutaneous lymphoma treated with palliative therapy. CR, complete remission; PR, partial remission; SD, stable disease; PD, progressive disease.

**Figure 4:**
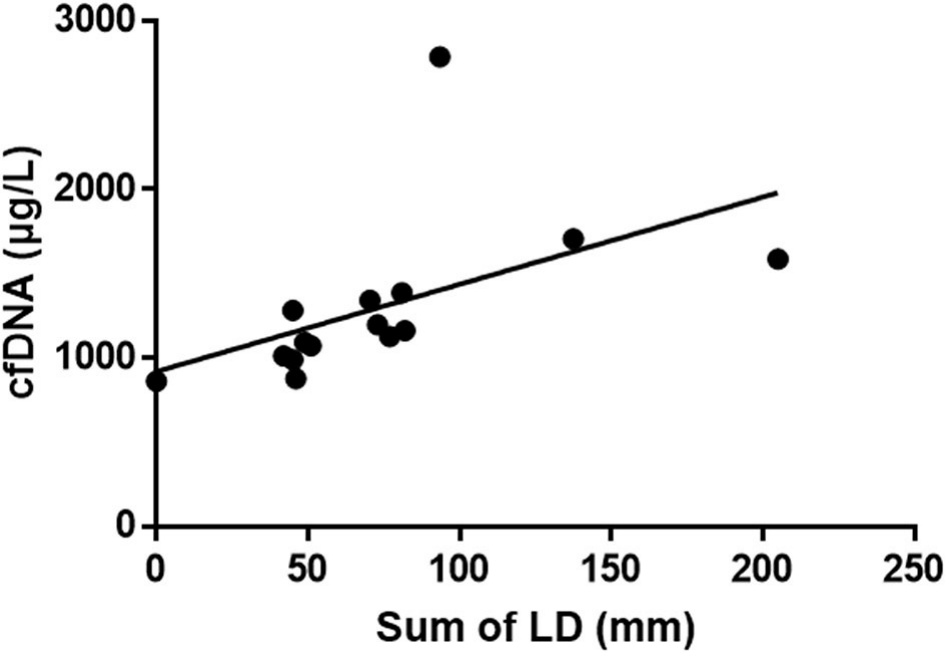
Correlation between the sum of lymph node diameter (LD) values and cell-free DNA (cfDNA) concentrations in dogs with multicentric lymphoma that had been treated using the L-CHOP protocol. Continual measurements of superficial lymph nodes and DNA concentrations reveal that the cfDNA concentration and the sum of LD are significantly positively correlated (Spearman correlation test, *R*^2^ = 0.26, *P* < 0.01).

The cfDNA concentrations were continually measured in six dogs with lymphoma (four *B*-cell origin and two *T*-cell origin). Two dogs showed complete remission (CR, all *B*-cell origin), whereas the other four dogs died of progressive disease. The median survival time (observation time) to present was 9 months (range, 3–14 months) in the *B*-cell lymphoma group and 7.7 months (range, 2 days−21 months) in dogs with *T*-cell lymphoma. The characteristics of individual dogs are presented in Supplementary Table 2, and the disease progression in each case is presented in Figure 3. Changes in cfDNA concentrations showed a similar pattern of response to chemotherapy. The cfDNA concentrations in two dogs after complete remission were lower than those before chemotherapy. Conversely, cfDNA concentrations in dogs with progressive disease or deceased dogs were increased. However, three out of five dogs that received chemotherapy showed a significant decrease in cfDNA concentration immediately after the first introduction. Two out of these five dogs showed complete remission, but the remaining three dogs were not responsive to the treatment. The overall sum of LD, the criterion used to monitor the response to chemotherapy, was significantly correlated with cfDNA concentrations (Spearman correlation test, *R*^2^ = 0.26, *P* < 0.01; Figure 4). Comparative analysis of the correlation between clinicopathological data and cfDNA was presented in Supplementary Table 3.

## Discussion

The present study investigated the utility of plasma cfDNA as a diagnostic, prognostic, and monitoring biomarker for neoplastic disease in dogs. Clinical data and plasma or serum samples derived from healthy, non-neoplastic, and neoplastic dogs with underlying diseases were compared to evaluate the role of this biomarker. According to our results, the mean cfDNA concentration in dogs with clinical illness was higher than that in healthy dogs, and cfDNA levels were significantly higher in dogs with neoplasms (particularly, lymphoid neoplasia) among clinically ill dogs. Based on a cfDNA cutoff value, it was found that high plasma cfDNA levels were associated with significantly lower survival rates. Furthermore, the treatment response was correlated with the cfDNA level. These findings suggest that cfDNA concentrations can be used to assess and monitor dogs with tumors.

Several hypotheses have been postulated to explain the origin of cfDNA in the bloodstream: (i) DNA leakage resulting from tumor necrosis or apoptosis, (ii) lysis of circulating cancer cells or micrometastases, and (iii) an unrevealed mechanism of active and spontaneous release (15, 16). Thus, changes in plasma cfDNA levels can reflect tumor burden, with cfDNA methylation, cancer-derived mutations, and loss of heterozygosity, which represent potential biomarkers for cancer detection (6). The role of cfDNA as an early diagnostic marker in human studies (4) focuses on disease screening through single sample collection and quantification of cancer-associated genetic alterations. In veterinary medicine, not many studies have been reported yet, but several studies have demonstrated the potential role of cfDNA in non-invasive early diagnosis. Some of these studies have focused on quantifying cfDNA levels to distinguish benign from malignant diseases (9, 17, 18). In the present study, plasma cfDNA concentrations were used to distinguish between clinically ill dogs and healthy dogs. The high cfDNA concentration in dogs with a clinically manifest illness is attributed to increased cell death from necrosis or apoptosis due to the underlying disease, resulting in an increased number of DNA fragments released into the circulation. Moreover, the cfDNA concentrations significantly increased in the order of healthy dogs, animals with an underlying non-neoplastic disorder, and dogs with a neoplastic disease. Given that the cfDNA concentration in the neoplastic group was significantly higher, it is likely that the elevated plasma cfDNA levels in this group were caused by increased necrosis of and DNA leakage from invasive tumor cells. In veterinary medicine, few studies have compared groups of clinically ill animals according to the presence or absence of tumors (6). Our report demonstrates significant differences in cfDNA concentrations between dogs with and without neoplasms. Additional follow-up studies are needed to more specifically assess the relationship between cfDNA concentration and survival time in each tumor type.

Significant differences in cfDNA levels based on the origin of the tumor could not be identified but compared to the healthy group, the group with lymphoid neoplasia had in this study high cfDNA concentrations. This is consistent with the results of a similar study (3), and previous studies found significantly elevated levels of plasma cfDNA in dogs with lymphoma, leukemia, and hemangiosarcoma compared to those with other malignancies (3, 6). In our study, the mean cfDNA concentration of 38 dogs with neoplasms was 1,435 μg/L. Of 10 dogs with lymphoma in the neoplastic group, 8 dogs had higher cfDNA levels than this mean cfDNA concentration, whereas 2 dogs had lower cfDNA levels. In addition, the DNA concentrations in the two dogs diagnosed with acute lymphocytic leukemia were with 2,770 μg/L and 2,285 μg/L at least 1.5-fold higher than the mean value of the entire neoplastic group, and this data represents that cfDNA concentration in the lymphoid neoplasia tends to be higher within the neoplastic group than in other types of tumor. These observations are consistent with the results of previous studies reporting that the cfDNA concentration is elevated in lymphoid neoplasia. The reason for the increased plasma cfDNA in dogs with lymphoid neoplasia is unclear but can be explained by cell viability. Necrosis, apoptosis, and cell fragility are frequently encountered in specimens collected from dogs with lymphoid neoplasms (19). The propensity for cell disruption may increase the leakage of tumor DNA into the circulation.

Previous studies in humans reported that plasma cfDNA levels are correlated with metastasis, larger tumor size, and higher cancer stage (20, 21). When the dogs with multicentric lymphoma were classified according to their clinical stage, the mean cfDNA concentration of two stage V dogs was 2,322.5 μg/L, whereas the mean concentration of the four stage IV dogs was 1,707.5 μg/L. This suggests a positive correlation between tumor stage and cfDNA concentration. However, since the number of dogs in this comparison was low, further prospective studies are warranted. Differences in cfDNA concentration according to tumor substage were not analyzed because all dogs with lymphoma had already been evaluated for clinical symptoms at the hospital, but cfDNA levels were compared according to the lymphoma immunophenotype. In dogs with lymphoma of *B*-cell and *T*-cell origin, the mean cfDNA concentrations were 1,594 μg/L and 2,055 μg/L, respectively. This may be explained by the shorter survival time and aggressive nature of CD3-positive canine lymphoma, and cell necrosis and consecutive release of cfDNA by the tumor might be more prevalent. It would be interesting to investigate in follow-up studies whether there are differences in cfDNA concentrations depending on the immunophenotype and whether this can predict the behavior of the tumor.

In our study, a cutoff value of the cfDNA concentration was determined in dogs with various types of tumors, and the survival rates were analyzed. Using a cfDNA concentration of 1,247.5 μg/L as the cutoff value for the 38 dogs of the neoplastic group, a significant difference (*P* = 0.024) in the survival rates of the two groups was found. This finding suggests that dogs with high plasma DNA had a significantly shorter survival (or observational) time, and a plasma cfDNA concentration of 1,247.5 μg/L may be a useful clinical cutoff value for the prognostic evaluation of these patients. Only a few studies in veterinary medicine have evaluated the prognosis of tumor patients using cfDNA levels. One study in dogs reported that regardless of the tumor type, cfDNA concentrations were able to distinguish tumors according to the presence or absence of metastasis (6). Another study determined the plasma cfDNA cutoff value in 19 dogs with lymphoid neoplasia for the evaluation of their remission time. This study demonstrates that dogs with high plasma cfDNA levels have a shorter remission time, whereas dogs with low cfDNA concentrations have a remission time longer than 10 weeks (3). Therefore, our study is the first report to analyze the survival rates of dogs with tumors based on their cfDNA levels. Additional follow-up studies are needed to more specifically assess the survival time for each tumor type. In conclusion, the increase in circulating cfDNA is of prognostic importance and associated with the short-term survival of patients.

Monitoring cfDNA levels can provide a snapshot of the disease progression. Detection of cfDNA is well-suited for real-time monitoring of cancer burden in response to therapy because of its short half-life and the reduced risk from repetitive liquid biopsies relative to imaging modalities or tissue biopsies (22). In humans, a variety of studies have reported decreased levels of cfDNA after tumor surgery and/or chemotherapy. Our study continually monitored the cfDNA level and its response to treatment in six dogs with lymphoma. Our results in dogs undergoing chemotherapy confirmed the negative correlation between prognosis and tendency to changes in cfDNA concentration at baseline. This finding is consistent with the higher levels of cfDNA in human cancer patients with poor treatment response, increased therapy resistance, higher risk of relapse, or reduced survival rate (17, 23). A significant discovery of our study is the correlation between the sum of LD and the cfDNA level of dogs with multicentric lymphoma; thus, continual monitoring of plasma cfDNA concentrations might be useful to assess the response to chemotherapy in patients with multicentric lymphoma. A recent study confirmed the correlation between cfDNA fraction (% of total plasma cfDNA) obtained through PCR for antigen receptor rearrangement (18) and the sum of LD during chemotherapy in four dogs with high-grade *B*-cell multicentric lymphoma. The molecular basis of the relationship between tumor burden and cfDNA levels was identified in this study. Moreover, an increase in cfDNA levels was observed 42 days before clinical relapse (e.g., an increase in lymph node size), confirming the potential of cfDNA measurements for monitoring tumor relapse (18). However, in our study, dogs no. 2 and 3 showed a sharp decrease in cfDNA concentration immediately before death. It remains unclear whether the lower cfDNA concentrations under these conditions were caused by bone marrow suppression, side effects of chemotherapy, or bone marrow infiltration by the tumor. Further studies are needed to elucidate the influence of metastatic changes on cfDNA concentrations at the cellular and molecular level.

A few limitations of this study must be mentioned. First, we could not compare prognosis after categorization based on the subtypes because of the low statistical power and various tumor types (multicentric vs. intestinal vs. cutaneous vs. hematogenous). Second, cfDNA is a very sensitive diagnostic biomarker for inflammation; therefore, it is difficult to completely rule out the possibility of elevated cfDNA levels in non-specific inflammation *in vivo*. Real-time PCR techniques with high specificity can be used to determine the underlying inflammatory mechanisms (24–27). Third, the observation period was short (up to 60 weeks), and each individual case had a different time frame. The analysis of the enrolled cases showed that the follow-up was censored along the way, resulting in limitations. Although the groups with high and low survival rates were significantly classified based on the determined cutoff value, it was difficult to calculate the expected survival time in these groups for this reason. It is desirable to analyze additional types of tumors over a longer period of time in a prospective study and determine the survival time according to the cfDNA level. Although any treatment did not significantly correlate with survival, the effect of different treatment protocols was not considered because of low statistical power. Lastly, a comparative analysis based on different chemotherapy protocols is required in further studies to examine the relationships of cfDNA concentrations with clinical outcomes of the protocols and drug resistance.

Our study is the first to describe a cutoff value for survival rates based on cfDNA concentrations in dogs with neoplastic disease. Furthermore, based on continual cfDNA monitoring of dogs with neoplasms, we identified the cfDNA concentration associated with poor clinical outcomes in dogs receiving chemotherapy. Overall, the results suggest that plasma cfDNA is a potentially useful indicator for monitoring treatment response and disease progression in dogs with tumors. Further large-scale studies are needed to determine the role of cfDNA as a surrogate biomarker in dogs with neoplastic diseases.

## Data Availability Statement

The original contributions presented in the study are included in the article/Supplementary Material, further inquiries can be directed to the corresponding author.

## Ethics Statement

The animal study was reviewed and approved by Gyeongsang National University. Written informed consent was obtained from the owners for the participation of their animals in this study.

## Author Contributions

JK, SA, and DY contributed to conception and design of the study. JK, HB, SS, and AC organized the database. JK and DY wrote the first draft of the manuscript. JK, D-IJ, K-WC, and DY wrote sections of the manuscript. All authors contributed to manuscript revision, read, and approved the submitted version.

## Funding

This research was supported by Basic Science Research Program through the National Research Foundation of Korea (NRF) funded by the Ministry of Science, ICT & Future Planning (2020R1C1C1008675). Funding sources had no involvement in study design, research conduct, or manuscript preparation.

## Conflict of Interest

The authors declare that the research was conducted in the absence of any commercial or financial relationships that could be construed as a potential conflict of interest.

## Publisher's Note

All claims expressed in this article are solely those of the authors and do not necessarily represent those of their affiliated organizations, or those of the publisher, the editors and the reviewers. Any product that may be evaluated in this article, or claim that may be made by its manufacturer, is not guaranteed or endorsed by the publisher.
